# Embryonic signature distinguishes pediatric and adult rhabdoid tumors from other SMARCB1-deficient cancers

**DOI:** 10.18632/oncotarget.15939

**Published:** 2017-03-06

**Authors:** Wilfrid Richer, Julien Masliah-Planchon, Nathalie Clement, Irene Jimenez, Laetitia Maillot, David Gentien, Benoît Albaud, Walid Chemlali, Christine Galant, Frederique Larousserie, Pascaline Boudou-Rouquette, Amaury Leruste, Celine Chauvin, Zhi Yan Han, Jean-Michel Coindre, Pascale Varlet, Paul Freneaux, Dominique Ranchère-Vince, Olivier Delattre, Franck Bourdeaut

**Affiliations:** ^1^ Paris-Sciences-Lettres, Institut Curie Research Center, INSERMU830 and SiRIC, Laboratory of Translational Research in Pediatric Oncology, Paris, France; ^2^ Paris-Sciences-Lettres, Institut Curie Hospital, Laboratory of Somatic Genetics, Paris, France; ^3^ Paris-Sciences-Lettres, Institut Curie Hospital, Department of Pediatric Oncology-Adolescents and Young Adults, Paris, France; ^4^ Paris-Sciences-Lettres, Institut Curie Research Center, Department of Translational Research, Genomic Platform, Paris, France; ^5^ University Hospital of Leuven, Department of Pathology, Leuven, Belgium; ^6^ Cochin University Hospital, Universite Rene Descartes, Sorbonne Paris Cite, Assistance Publique Hôpitaux de Paris, Department of Pathology, Paris, France; ^7^ Cochin University Hospital, Assistance Publique Hôpitaux de Paris, Department of Oncology, Paris, France; ^8^ Institut Bergognie, Department of Pathology, Bordeaux, France; ^9^ University Sainte-Anne Hospital, Paris, France; ^10^ Paris-Sciences-Lettres, Institut Curie Hospital, Department of Pathology, Paris, France; ^11^ Centre Leon Berard, Department of Pathology, Lyon, France

**Keywords:** rhabdoid, SMARCB1, TET1, DNMT3B, adult

## Abstract

Extra-cranial rhabdoid tumors (RT) are highly aggressive malignancies of infancy, characterized by undifferentiated histological features and loss of SMARCB1 expression. The diagnosis is all the more challenging that other poorly differentiated cancers lose SMARCB1 expression, such as epithelioid sarcomas (ES), renal medullary carcinomas (RMC) or undifferentiated chordomas (UC). Moreover, late cases occurring in adults are now increasingly reported, raising the question of differential diagnoses and emphasizing nosological issues. To address this issue, we have analyzed the expression profiles of a training set of 32 SMARCB1-deficient tumors (SDT), with ascertained diagnosis of RT (*n* = 16, all < 5 years of age), ES (*n* = 8, all > 10 years of age), UC (*n* = 3) and RMC (*n* = 5). As compared with other SDT, RT are characterized by an embryonic signature, and up-regulation of key-actors of de novo DNA methylation processes. Using this signature, we then analysed the expression profiling of 37 SDT to infer the appropriate diagnosis. Thirteen adult onset tumors showed strong similarity with pediatric RT, in spite of older age; by exome sequencing, these tumors also showed genomic features indistinguishable from pediatric RT. In contrary, 8 tumors were reclassified within carcinoma, ES or UC categories, while the remaining could not be related to any of those entities. Our results demonstrate that embryonic signature is shared by all RT, whatever the age at diagnosis; they also illustrate that many adult-onset SDT of ambiguous histological diagnosis are clearly different from RT. Finally, our study paves the way for the routine use of expression-based signatures to give accurate diagnosis of SDT.

## INTRODUCTION

Rhabdoid tumors (RT) have been initially described as rare morphological variants of Wilms tumors, characterized by the presence of rhabdoid cells in aggressive tumors occurring in infants. They were then described in soft-parts and, eventually, brain tumors where they are referred to as “Atypical Teratoid Rhabdoid Tumors” (AT/RT). The genetic hallmark of all rhabdoid tumors is the biallelic inactivation of *SMARCB1* tumor suppressor gene [1]. *SMARCB1* inactivation is an almost constant hallmark but it has also been demonstrated to be the only recurrent mutation encountered in RT, which show the most stable genome among human malignancies [2]. Although the cell of origin is not identified yet, some arguments suggest that RT may derive from progenitors or pluripotent cells [3–5]. Altogether, RT could be defined as highly aggressive tumors, potentially deriving from early progenitors, occurring in infants and young children, and driven by *SMARCB1* biallelic inactivation as the sole and unique genetic event.

*SMARCB1* encodes a ubiquitously expressed core member of the SWI/SNF complex, involved in ATP-dependent chromatin remodeling. Next-generation Sequencings have revealed that mutations in one or another member of the SWI/SNF complex affect about 20% of human malignancies [6], enlightening that RT is a founder member of a large family of cancers, biologically-defined by SWI/SNF deficiency. In the past few years, several studies have broadly expanded the spectrum of tumors that show a *SMARCB1* loss of expression, with or without documented genetic alterations, many of which arise in adults [7]. This SMARCB1-deficient family now comprises some clear nosological entities, such as epithelioid sarcomas (ES) [8–10], renal medullary carcinomas (RMC) [11], undifferentiated chordomas (UC) [12, 13], or epithelioid malignant peripheral nerve sheath tumors (eMPNST) [7]; it also encompasses less defined tumors and nosological entities that may be hard to diagnose or distinguish from aforementioned malignancies. Immunomarkers are sought for to help pathologists distinguishing these entities, but molecular approaches may be added to provide useful diagnostic tools.

An increasing number of SMARCB1-deficient tumors with rhabdoid phenotype, and therefore named “RT”, are now reported in adults. Whether these tumors should be considered as a late occurrence of pediatric-like RTs, as a distinct entity, or as misdiagnosed other adult-type *SMARCB1*-deficient tumors, remains to be elucidated. In this manuscript, we report the expression profile of a large series of late-onset SMARCB1-deficient tumors of uncertain diagnosis and demonstrate that most of those tumors considered as “RT” significantly differ from their pediatric counterparts. We finally aim to provide simple molecular signatures that may be implemented in routine diagnosis procedures in a close future.

## RESULTS

The diagnosis of RT is hard to ascertain when histological, clinical and genetic features are not all typical for that diagnosis. In order to base our comparisons on a robust dataset, we decided to first analyse a “training set” composed by tumors for which all criteria, i.e. histological, clinical and genetic features converged to ascertain the diagnosis of RT. We then analysed a series of samples with uncertain diagnosis (thereafter referred to as “study cohort”) based on the results obtained from the training set.

### Expression profiling distinguish RT from all other SD-NRT

**Figure 1:**
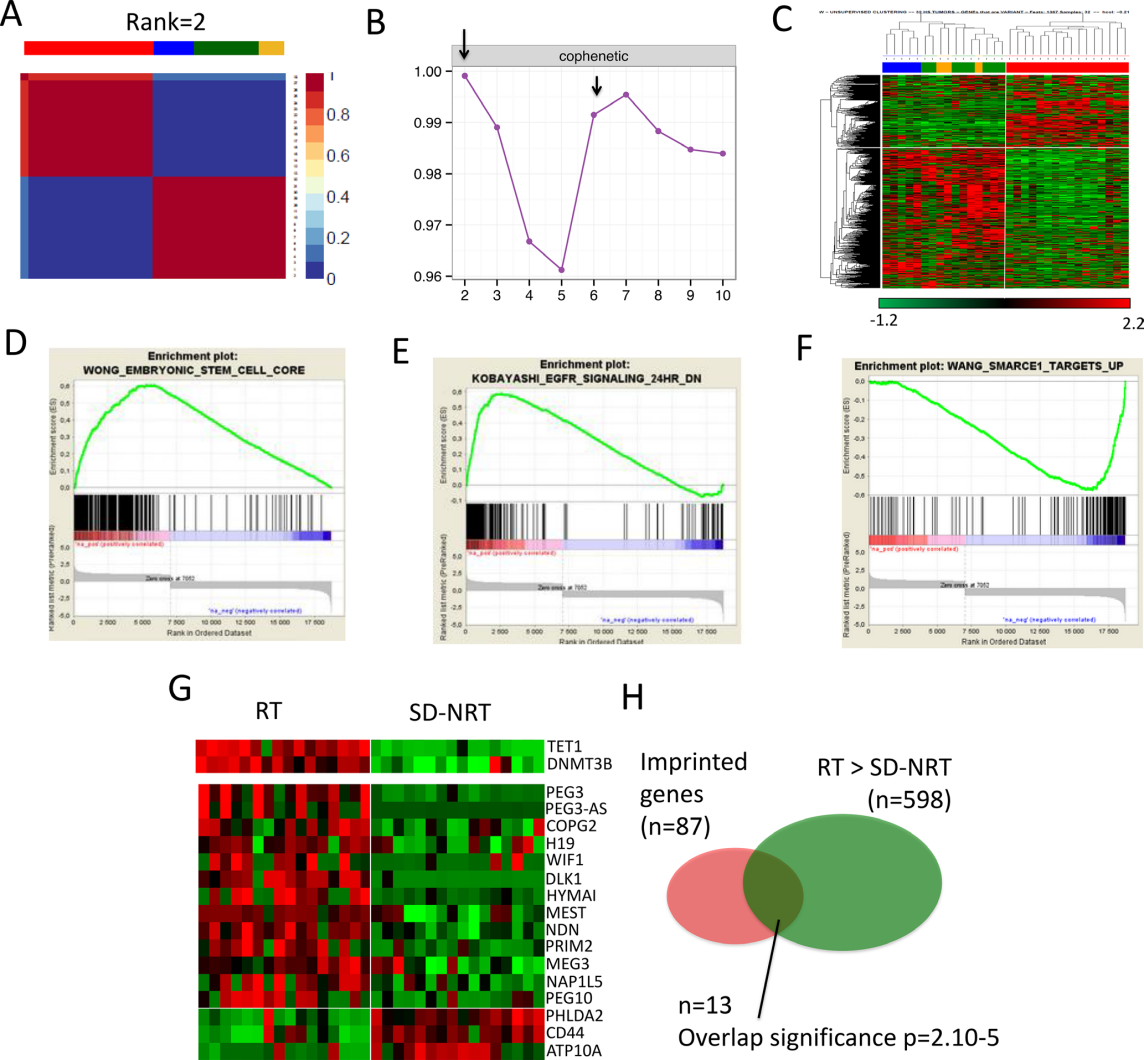
Comparisons between training set Rhabdoid tumors (RT) and training set SMARCB1-deficient non rhabdoid tumors (SD-NRT) (**A**) Non Matrix factorization (NMF) performed on the 32 tumors and (**B**) cophenetic scores, showing that RT and all others SD-NRT first clusterize in two main subgroups; RT is a clearly distinct entity (**C**) Similar results obtained by unsupervised hierarchical clustering (**D, E, F**) Gene Set Enrichment Analyses showing 3 biological signatures that distinguish RT from SD-NRT, (**G**) heat-map showing the relative expression of TET1 and DNMT3B (upper panel) and imprinted genes (lower panels) in RT vs SD-NRT. (**H**) Venn diagram showing the significance of the over-representation of imprinted genes in the list of differentially expressed genes.

We first analysed the transcriptomes of SMARCB1-deficient tumors with ascertained diagnoses of RT (*n* = 16), and SMARCB1-deficient non rhabdoid tumors (SD-NRT, *n* = 16) with ascertained diagnosis of epithelioid sarcomas (ES, *n* = 8), renal medullary carcinoma (RMC, *n* = 5) and undifferentiated chordomas (UC, *n* = 3); these tumors constituted the “training set” (see material and methods, clinical and genetic features in Table 1). To assess the actual differences between these tumor types, we first applied two orthogonal unsupervised clustering methods, i.e. non-negative matrix factorization (NMF) (Figure 1A, 1B) and unsupervised hierarchical clustering (Figure 1C). Both methods robustly split our cohort in two obvious groups, i) group 1, composed of 16/16 histologically defined RT and, ii) group 2: composed of 16/16 histologically defined SD-NRT. Thus, all SD-NRT, whatever their histological type, clustered apart from RT, which in turn constituted a robust isolated entity. Of note, the cophenetic correlation also indicated that these two main and obvious groups could be divided in up to 6 to 7 sub-entities (Figure 1B). Consistently, hierarchical unsupervised clustering also suggested the existence of sub-groups within the two main branches, reflecting the diversity of both the RT and the SD-NRT (Figure 1C). These results were consistent with the known diversity within SD-NRT.

**Table 1:** Training set, clinical and genetic features

	Age	Location	*SMARCB1* First hit	*SMARCB1* Second hit	Diagnosis
**RT**					
INI18	<2	Kidney	Del ex4-5	Del ex4-5	RT
INI19	0.4	Soft-parts	Del ex6	Del ex6	RT
INI22	4.5	Soft-parts	Del ex1-9	Del ex1-9	RT
INI23	<2	Kidney	ND	ND	RT
INI24	0.1	Soft-parts	c.472C > T (p.Arg158*)	LOH	RT
INI26	0.5	Soft-parts	Del ex1-9	Del ex1-9	RT
INI39	1	Kidney	c.157C > T (p.Arg53*)	LOH	RT
INI50	0.1	Soft-parts	Del ex1-9	Del ex1-9	RT
INI59	0.5	Soft-parts	c.601C > T (p.Arg201*)	LOH	RT
INI56	0.1	Soft-parts	Del ex1-9	Del ex1-9	RT
INI90	1.8	Bladder	Del ex1-9	Del ex1-9	RT
INI91	2.1	Brachial Plexus	Del ex1-9	Del ex1-9	RT
INI93	2	Kidney	Del ex1-9	Del ex1-9	RT
INI97	1.9	Kidney	c.950del (p.Gly317Aspfs*3)	LOH	RT
INI109	1.7	Soft-parts	Del ex1-9	Del ex1-9	RT
INI110	2.5	Soft-parts	Del ex1-9	Del ex1-9	RT
**SD-NRT**					
INI85	38	Kidney	Del ex1-9	translocation	SD-NRT (RMC)
INI95	29	Kidney	Del ex1-9	translocation	SD-NRT (RMC)
INI111	8	Kidney	Del ex1-9	Del ex1-9	SD-NRT (RMC)
INI137	16	Kidney	Del ex1-9	translocation	SD-NRT (RMC)
INI141	33	Kidney	Del ex1-9	translocation	SD-NRT (RMC)
INI138	2.2	Clivus	Del ex1-9	Del ex1-9	SD-NRT (UC)
INI142	2	Clivus	Del ex1-9	Del ex1-9 ?	SD-NRT (UC)
INI144	3	Clivus	Del ex1-9	Del ex1-9 ?	SD-NRT (UC)
PT25	48	Pelvis	Del ex1-9	Del ex1-9	SD-NRT (ES)
PT26	13.6	Groin	ND	ND	SD-NRT (ES)
INI66	23	Thigh	Del ex1-9	Del ex1-9	SD-NRT (ES)
INI121	29	Thigh	ND	ND	SD-NRT (ES)
INI122	25.8	Forearm	ND	ND	SD-NRT (ES)
INI124	17	Perineum	ND	ND	SD-NRT (ES)
INI125	18	NA	ND	ND	SD-NRT (ES)
INI126	16.8	Arm	Del ex1-9	Del ex1-9 ?	SD-NRT (ES)

Age in years. Del: deletion. ND: not done. NA: not available

### RT are characterized by an embryonic signature and indirect clues for imprinting defects

To delineate a signature for RT as compared with SD-NRT, we used two parallel methods, i.e., i) a Welch *t-test* comparing RT with all other SD-NRT (Supplementary Table 2), and then RT with each other individual SMARCB1-deficient tumor group (pair-wise analysis); a heat map of the 20 top genes for each group is depicted in Supplementary Figure 1; and ii) a NMF-based list, focused on the 20% most differential genes defined by the NMF method; this resulted in a short list of 161 genes, 50 overexpressed in RT and 111 overexpressed in SD-NRT (Supplementary Table 3).

NMF-based gene list showed an overexpression of embryonic stem cell genes such as *SALL2*, *LIN28B* and the *FGF* pathway (*FGFR2, FGF9, FGFBP3*). The sonic hedgehog inhibitor *HHIP* was also overexpressed. *TET1* and *DNMT3B*, two genes involved in imprinting erasure and *de novo* DNA CpG methylation, respectively, in germinal and embryonic stem cells, also showed up in RT compared with other SD-NRT (Figure 1G, Supplementary Table 2 and Supplementary Figure 1).

The Welch *t-test* comparisons between RT and all SD-NRT identified 598 genes that were significantly overexpressed in RT as compared with all SD-NRT; 157/161 of the NMF based signature were included in this wider list. DAVID analyses (Supplementary Table 2) and Gene Set Enrichment Analyses (GSEA, Figure 1D) revealed that pathways that characterized RT in comparison with SD-NRT were related to cell cycle, morphogenesis, and embryonic development. EGFR signaling characterized RT (Figure 1E), while some SWI/SNF targets characterized SD-NRT (Figure 1F). Looking to genes in details, RT were again characterized by a specific overexpression of *FGF* pathway (*FGFR2, FGF9* and *FGFBP3*), *SALL2* and *SALL4* genes, *HHIP* and its antagonistic lncRNA *HHIP-AS*. *HOXC* genes cluster (Supplementary Figure 1), and *HOTAIR* (Supplementary Table 2) were up-regulated in RT, in agreement with the known role of *HOTAIR* in regulating *HOXD* and *HOXC* genes. Finally, *TET1* and *DNMT3B* also showed up in RT. The overexpression of two genes involved in imprinting mechanisms prompted us to investigate whether imprinted genes were subject to significantly aberrant expression in RT. Based on the list of imprinted genes published by Morison et al. [14], we indeed found a significant over-representation of imprinted genes in the list of genes overexpressed in RT (Figure 1G, 1H).

Altogether, RT were characterized by an embryonic stem cell signature, indirect clues in favor of DNA imprinting disorder and an overexpression of genes involved in *de novo* imprinting regulation, such as *TET1*. These results were confirmed by RT-PCR for a subset of significant genes (Supplementary Figure 2).

### Characteristics of the study cohort

Among the 69 included tumors, 37 were considered of uncertain diagnosis by pathological analysis and constituted the “study cohort” (Clinical and genetic features are reported in Tables 2 and 3). This comprised: i) SMARCB1-deficient tumors occurring in children younger than 5 years with no central review (*n* = 5), ii) SMARCB1-deficient tumors occurring in children younger than 5 years, with central review but showing no rhabdoid morphology (*n* = 2), and iii) SMARCB1-deficient tumors arising in patients older than 5 years without central review or with central review but diagnosed neither RT, nor ES, nor RMC nor UC (*n* = 30). In those latter 30 patients, the diagnosis of RT was evoked by local pathologists in 23 cases, while no specific diagnosis was initially retained for 7 cases.

**Table 2:** Study cohort, clinical features

	Location	Age	Sex	Outcome	Follow-up (days)	Initial>Corrected
**RT**						
INI25	Soft-part	0.2	M	NA	NA	RT>RT
INI29	Kidney	?	M	NA	NA	RT>RT
INI44	Kidney	7.3	M	NA	NA	RT>RT
INI53	Periph. nerve	0.7	M	NA	NA	RT>RT
INI61	Thx, pararachis	8	M	NED	1825	RT>RT
INI64	Forearm	21	M	DOD	300	RT>RT
INI105	Retro-periton.	33	F	DOD	66	RT>RT
INI116	Foot	3.4	M	NED	1370	RT>RT
INI120	Paraspinal/ORL	18	F	DOD	774	RT>RT
INI127	Groin	40	M	NED	790	ES>RT
INI135	Fro. meninges	22	M	NED	60	RT>RT
INI136	Brachial plexus	14	M	NED	1005	RT>RT
INI143	Kidney	10	M	NED	610	RT>RT
INI174	Soft-part, limb	27	M	DOD	172	MyoEC >RT
INI176	Soft-part, leg	28	F	NA	NA	RT>RT
INI185	Vulva	43	F	NA	NA	MyoEC>RT
**SD-NRT**						
INI20	Adrenal Gland	6.7	M	DOD	120	RT> SD-NRT
INI21	Kidney	1.6	F	DOD	179	RT>SD-NRT
INI37	Lung	25	F	DOD	51	RT>ES
INI38	Bladder	0.8	M	DOD	90	RT> SD-NRT
INI65	Periph. nerve	54	F	DOD	93	eMPNST>SD-NRT
INI86	Thorax	6	M	NED	13yrs	RT>SD-NRT
INI106	Soft-parts	1.2	M	NED	988	RT>SD-NRT
INI114	Thorax	26	F	DOD	387	RT> SD-NRT
INI115	Cavum	26	F	NED	855	SD-NRT>SD-NRT
INI117	Paraspinal	16	M	DOD	122	RT>UC
INI123	Not found	38	F	DOD	260	ES>SD-NRT
INI128	Leg	13	M	NED	422	ES>ES
INI129	Groin	53	F	*NA*	*NA*	SD-NRT>SD-NRT
INI130	Buttock	9	F	NED	810	ES>ES
INI131	Pleura	31	M	DOD	155	RT>SD-NRT
INI132	Para. meninges	39	M	NED	239	AT/RT>SD-NRT
INI133	Elbow	10	M	NED	684	ES>ES
INI134	Abdomen	34	M	DOD	7	RT>ES
INI152	Periph. nerve	30	F	DOD	45	MPNST> SD-NRT
INI175	Neck	14	F	DOD	910	RT>ES
INI182	Kidney	51	M	NED	-	RT>SD-NRT

M: male. F: female. Age in years NED: no evidence of disease. DOD: dead of disease. *NA*: not available. Follow-up in days. “Initial>corrected” indicates the local histological diagnosis and the molecular diagnosis, respectively. MyoEC: myoepithelial carcinoma. MPNST: malignant peripheral nerve sheath tumor. Periph: peripheral. Thx: thorax. Fro: frontal. Retro-periton.: retroperitoneum.

**Table 3:** Study cohort, genetic features

	SMARCB1 first hit	SMARCB1 second hit	Variants in known cancer genes
**RT**			
INI25	ND	ND	ND
INI29	ND	ND	ND
INI44	Del Ex1-5	Del Ex1-9	ND
INI53	Del Ex1-9	Del Ex1-9	ND
INI61	Del Ex1-9	c.243C>G (p.Tyr81*)	ND
INI64	c.321C>A (p.Tyr107*)	Del Ex1-2	*CHD7*: c.2238+1G>A
INI105	Del Ex1-9	c.211_212insGATACACAACA (p.Lys71Argfs*18)	*CHD5*: p.R966Q
INI116	Del Ex1-9	Del Ex1-9	ND
INI120	Del Ex1-9	Del Ex1-9	ND
INI127	c.157C>T (p.Arg53*)	LOH	ND
INI135	c.618G>A (p.Trp206*)	c.832C>T (p.Gn278*)	ND
INI136	Del Ex1-9	Del Ex1-9	None
INI143	c.152G>A (p.Trp51*)	LOH	*POLE*: c.604+2T
INI174	c.94-1G>A (p.?)	Del Ex6	None
INI176	c.152G>A (p.Trp51*)	Del Ex1-9	*NTRK2*: p.P204H
INI185	Del Ex1-9	Del Ex1-9	ND
**SD-NRT**			
INI20	Del ex1-6	Del ex1-9	ND
INI21	c.157C>T (p.Arg53*)	Del ex1-9	ND
INI37	Del ex1-9	Del ex1-9	ND
INI38	Del Ex1-9	Del Ex1-9	ND
INI65	Del ex1-9	Del ex1-9	*ATM*: p.F858L
INI86	Del ex1-9	Del ex1-9	ND
INI106	Del ex1-9	Del ex1-9	ND
INI114	Del ex1-7	Translocation	*ARID1A*:p.G180fs
INI115	Del Ex1-9	Del Ex1-9	ND
INI117	Del Ex1-9	Del Ex1-9	*DDX53*:p.S275C *WNT8A*: p.D134N
INI123	ND	ND	ND
INI128	Del Ex1-9	Del Ex1-9 ?	ND
INI129	Del Ex1-9	Del Ex1-9	ND
INI130	ND	ND	ND
INI131	c.544C>T (p.Gln182*)	c.592_613delinsTGCCTTCC (p.Gln198Cysfs*8)	ND
INI132	Del ex1-9	Del ex2-9	*NRAS*: p.G12D
INI133	Del Ex1-9	Del Ex1-9 ?	ND
INI134	p.Gly80*(c.238G>T)	c.564del (p.Ile189Serfs*20)	ND
INI152	c.580G>T (p.Glu194*)	LOH	*ATM*: p.F858L *NSD1*: p.T2029A
INI175	ND	ND	ND
INI182	Del Ex1-9	Del Ex1-9	*ETV4*: p.R160C *ID2*: p.L106F

Del: deletion. ND: not done. LOH: loss of heterozygosity.

### Some late-onset SMARCB1-deficient tumors are very similar to pediatric cases

**Figure 2:**
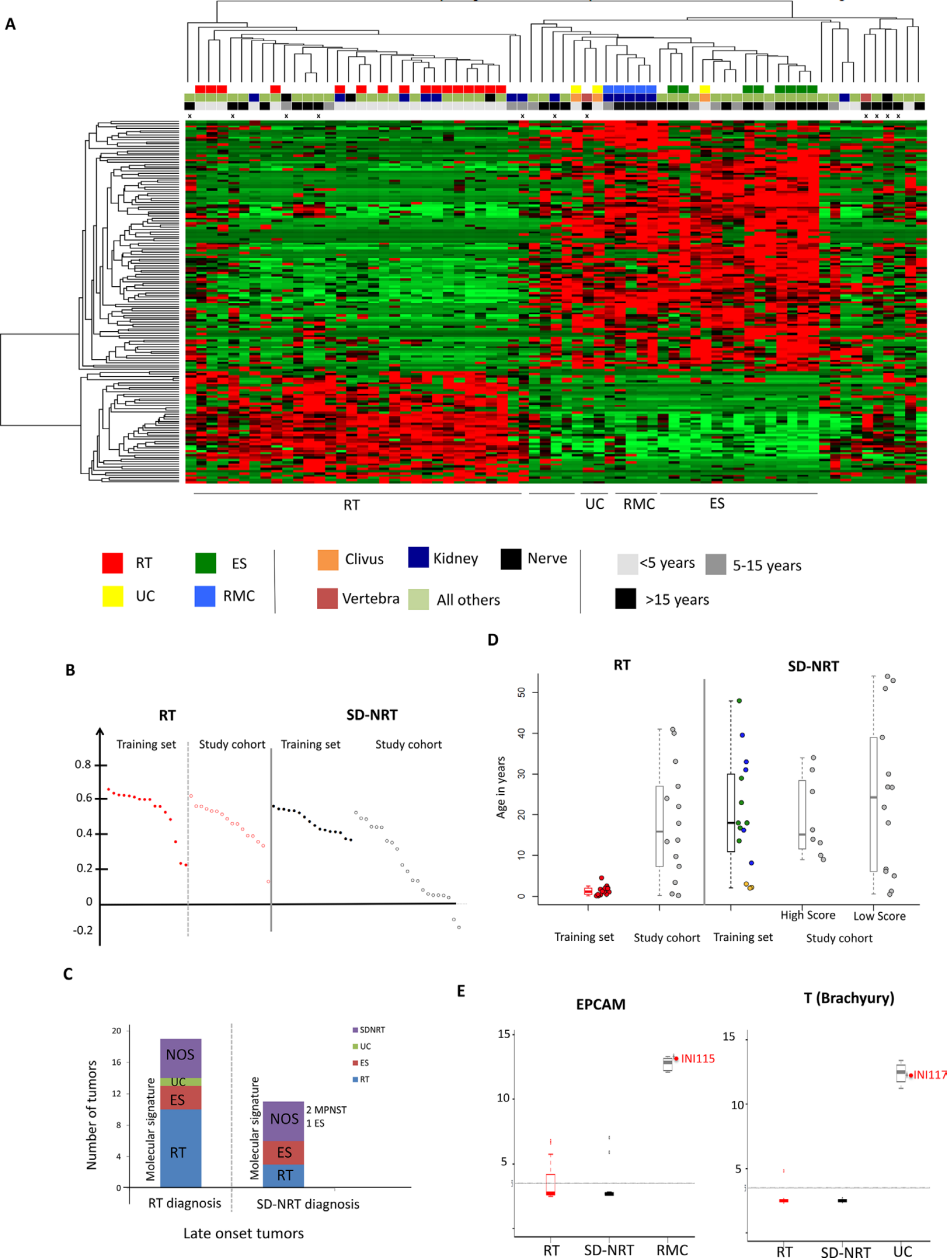
Expression profiles on a series of 64 SMARCB1-deficient cancers helps to assign the appropriate diagnosis (**A**) Clustering of 69 SMARCB1-deficient cancers on the gene set characterizing RT and SD-NRT according to NMF including the training set: rhabdoid tumors, “RT”, red squares of the first line; non-rhabdoid tumors, i.e. renal medullary carcinomas (“RMC”, blue squares), epithelioid sarcomas (“ES”, green squares) and undifferentiated chordomas (“UC”, yellow squares). Second line, anatomic location of the tumors: orange square: clivus; brown squares: vertebra; blue square: kidney; black square: peripheral nerve; pale green: all other locations. Third line: age at tumor diagnosis: black square: above 15 years old; dark grey: between 5 and 15 years old; pale grey: below 5years old. Fourth line: crosses indicate samples for which whole genome DNA copy number and/or sequencing were assessed. (**B**) Silhouette score for each sample, in the RT cluster and the SD-NRT cluster. Silhouette scores for training set tumors are plotted in dark red and black for RT and SD-NRT, respectively; Silhouette scores for study set tumors are plotted in empty red and empty black for RT and SD-NRT, respectively. (**C**) Molecular re-classification of each sample according to the clustering; “RT diagnosis” and “SD-NRT diagnosis” refer to the diagnosis initially proposed by the local pathologist. (**D**) age distribution in each group of cancers; in box plots, the central rectangle spans the first quartile to the third quartile (interquartile range or IQR); the horizontal line inside the rectangle shows the median; whiskers are taken to 1.5 times the IQR range from the box; circles show outliers. Each plot corresponds to one sample. (**E**) Expression of one carcinoma (EPCAM) and one chordoma (Brachyury, “T”) markers indicating specific pathological subtypes for at least two unclassified SD-NRT (INI115 and INI117).

In an attempt to classify each tumor of the study cohort (*n* = 37) to one of the categories defined on the training set, we performed a supervised clustering using the NMF-based gene list (defined in the second paragraph) on all 69 tumors (Figure 2A); we then estimated how robustly each sample was allocated to its cluster using the Silhouette algorithm (Figure 2B).

On this basis, 16/37 study cohort tumors clustered with training set RT; 15/16 showed a Silhouette score at least equivalent to the scores of training set RT, suggesting that these tumors actually behave as true RT. All were diagnosed as RT by the pathologists (Figure 2C); 7 patients were older than 15 years of age and 4 were between 5 and 15 years of age (Figure 2D).

### Late-onset SD-NRT disctinct from RT

Then, 21/37 study cohort tumors clustered outside the training set RT group (Figure 2A) 8/21 showed a Silhouette score at least equivalent to those of training set SD-NRT, suggesting that they were undiagnosed ES, RMC or UC (Figure 2B). In details, six clustered more closely with ES, retrospectively suggesting this diagnosis; this comprised 3 late pediatric cases (9, 10 and 13 years) in which RT and ES diagnoses were discussed because of the unusual age for both tumor types. One tumor from the gut showed some similarity with RMC and high expression of *EPCAM*, an epithelial and carcinoma marker (Figure 2E). Another tumor (INI117, paravertebral, > 15 years) clustered with UC and showed a high expression of *T* (Brachyury, a marker of chordoma), retrospectively suggesting a diagnosis of UC (Figure 2E).

In contrary, 13/21 showed a weak Silhouette score. This suggested that these last tumors were definitely not RT, but behaved also differently from ES, RMC and UC, being either known SMARCB1-deficient entities not included in our training set (eMPNST, extra-skeletal chondrosarcomas, etc…), or unknown other entities. Six were nevertheless diagnosed as RT by the local pathologists.

### Adult and pediatric RT show similar patterns

Among the 16 study cohort tumors clustering with training set RT, 13 were late-onset tumors (> 5 years; median age, 18 years). When RT were strictly defined by the belonging to the RT group according to these clustering methods, the Silhouette score didn't significantly correlate or anti-correlate with the age at diagnosis (r = 0.15, Supplementary Figure 3). Thus, late-onset RT, provided that they were selected based on a specific gene expression signature, behaved very similarly to their early-onset counterparts. In particular, we looked at the expression pattern of the embryonic cell genes previously identified; these late-onset RT showed similar pattern of expression of imprinted genes (Supplementary Figure 3B), *HOTAIR* and *HOXC* genes cluster (Supplementary Figure 3C), and embryonic genes. Altogether, our data suggested that a substantial number of late-onset SMARCB1-deficient considered as RT by pathologists don't significantly differ from pediatric cases.

### Genomic landscape of late-onset RT

**Figure 3:**
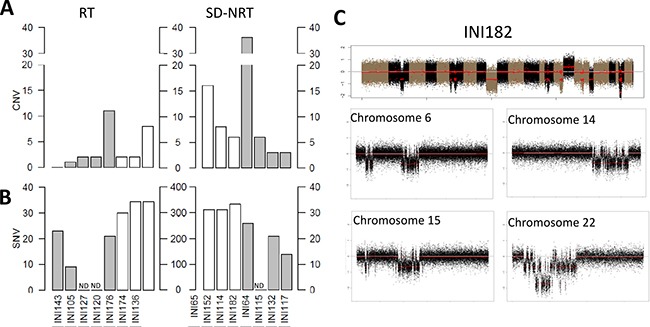
Genomic features of late-onset SD-NRT Numbers of (**A**) Copy number variations (CNV) and (**B**) single nucleotide variants observed in tumors belonging to (**C**) the “RT” group (left panel) and to the “SD-NRT” group (right panel). Grey bars indicate tumors (A, B) for which both tumor and germline DNA could be analysed and numbers of SNV (b) are indicated by the scales that range from 0 to 40. White bars indicate tumors for which no germline DNA could be analysed and numbers of SNV (B) are indicated by the scales that range from 0 to 400. ND: not done. (C) CNV profile on tumor 182, harboring chromothripsis features on multiple chromosomes, including chromosome 22 that shows a homozygous deletion at *SMARCB1* locus.

Beyond their embryonic features, RT are characterized by a remarkably simple genome, with no recurrent variants apart from *SMARCB1*. In order to illustrate whether late-onset RT displayed the same profile, we performed exome sequencing on 5 samples and copy number genome wide analysis on another 2 samples (Figure 3). Altogether, Copy Number Variation (CNV) count was remarkably low (median = 2). Apart from *SMARCB1*, no gene was recurrently found to be mutated in late-onset RT. Seven somatically acquired Single Nucleotide Variants (SNV) predicted to be deleterious was found in INI105, and 23 in INI143, the only two tumors for which the germline DNA was available (Supplementary Table 4). No gene was recurrently mutated among late-onset RT. Of note, a variant of *POLE2*, a gene involved in genomic stability, was found in INI143, which nevertheless harbored a low mutation load. Altogether, late-onset RT showed few genomic abnormalities within an overall stable genome, and no recurrent mutation apart from *SMARCB1*.

In order to compare these features with other late-onset SMARCB1-deficient cancers, we assessed the genomic landscape of 8 SD-NRT from the study cohort, 7 by exome sequencing, and one by array-CGH. The median number of CNV was 7 (3 to 36), with one sample showing multiple breakpoints on few chromosomes, suggestive of chromothripsis (IN165, Figure 3C). In this sample, *SMARCB1* locus was comprised in the chromothripsis of chromosome 22q, thus inactivated by a mechanism unusually found in RT. Tumors for which the germline DNA was available (INI65, INI114 and INI152) showed 14, 21 and 26 somatically acquired SNV (reported in Supplementary Table 3). Again, no recurrent mutation was found. Of note, one deleterious frameshift mutation affecting *ARID1A* exon 1 was found in one tumor in addition to *SMARCB1* loss.

### Outcome of late-onset RT

Altogether, clinical outcome was available for 16 patients with an adult-onset (>15 years at diagnosis) SMARCB1-deficient tumor, including 6 RT and 10 SD-NRT. With a median follow-up of 236 days, 4/6 patients inferred to have a late-onset RT died of disease; the latency between first symptoms and diagnosis, which may reflect tumor aggressiveness, ranged from 3 weeks to one year (Supplementary Table 1). 7/10 patients inferred to have a SD-NRT died of disease, with a median follow-up of 155 months; the latency ranged within similar time lapses (Supplementary Table 1). Altogether, adult-onset RT did not strikingly differ from other adult onset SMARCB1-deficient cancers from our series, all categories having a rather poor outcome.

## DISCUSSION

The definition of the “rhabdoid tumor” entity is confusedly based on the presence of a phenotype “compatible with this diagnosis” in a context of *SMARCB1* bi-allelic inactivation and/or BAF47 loss of expression. However, the expansion of the *SMARCB1*-deficient family of cancers and the pleomorphic morphology of RT has definitely challenged this simplistic definition. The main clinical perspective of our work is to find some biological features that may help pathologists to classify SMARCB1-deficient cancers in the appropriate entity. Specific markers for immunohistochemistry have been looked for, with variable success. Kohashi et al. [15] and Yoshida et al. [16] both found SALL4 to be a specific marker for RT when compared with ES; however, the protein was not constantly expressed in all RT, underscoring the weakness of single marker-based diagnoses. Genomic profiling may provide another tool for diagnostic orientation. Our study exemplifies that true RT have a remarkably stable genome, whatever the age at diagnosis. However, this may not be specific to RT among *SMARCB1* deficient cancers [11]. In contrary, tumors with several CNV are unlikely to be true RT. Complex genomic profiles have already been reported in ES [10], and may be shared by other SD-NRT entities.

In addition to the genomic profile, expression profile-based signature may provide potent tools for tumor classification. Kohashi et al. [15] and Sapi et al. [17] both used miRNA expression profiling to distinguish ES from other SD-NRT; both studies identified different but not fully convergent patterns of miRNA expression in ES and RT. Although these studies shed some light on new mechanisms for *SMARCB1* down-regulation, they did not inform on the biological origin of the tumors. In the present study, we assumed that comparing “true” RT with “true” SD-NRT could give insights on the actual identity of RT. Our results suggest that RT are characterized by an embryonic signature, and that this is true whatever age at diagnosis. Interestingly, the overexpression of *HOX* genes clusters and the negative regulator of HOXC, *HOTAIR*, is now consistently observed in several independent series [18–20]. Another interesting observation is the specific overexpression of *TET1* and *DNMT3B* regulators of DNA methylation. *TET1* is a key enzyme responsible for active DNA demethylation by converting 5-methyl cytosine to 5-hydroxymethylcytosine, a first step towards full demethylation [14, 21]. A globally hypermethylated pattern has recently been reported in RT [19, 20], but the precise kind of methylation (hydroxyl-methyl or methyl cytosine) was not assessed. Hypermethylation may be linked, in contrary, to DNMT3 proteins, a methyltransferase family that increase the global DNA methylation [22]. However, DNMT3 recruitment to intergenic differentially methylated regions is altered by PRC2 complex [23]. Given that *SMARCB1* loss releases EZH2 activity [5], it is likely that DNMT3B recruitment and subsequent DNA methylation may be impaired by PRC2 overfunction in RT. The role of a dynamic balance between TET1 methylation erasure, PRC2 overactivity and DNMT3B methyltransferase overexpression would now require deeper investigation, assessing the balance between methylcytosine versus hydroxymethylcytosine methylation in RT.

One recent study suggested the existence of two different types of extra-cranial rhabdoid tumors, based on miRNA, expression, methylation, exome and ChIP-Seq analyses [19]. Our NMF analyses on the training set rather strikingly showed that RT constitute a consistent group as compared with SD-NRT. However, the cophenetic scores peak at 7 (Figure 1B) suggested some diversity, not only within the SD-NRT group, but also within the RT group. This diversity didn't lead to identify significant subgroups in our analysis, possibly due to small numbers. Alternatively, differences between putative RT subgroups may be too subtle, and not as relevant as differences evidenced between various SMARCB1-deficient cancers, for which our differential analyses were designed. Eventually, while our study brings evidence for clear discrimination between RT and other SD-NRT, appropriate subgrouping within RT will be all the more relevant in clinics that it actually allows treatment stratification, which still requires some more evidence.

Finally, our study for the first time in some details describes adult-onset RT. Although the presence of rhabdoid features is frequently depicted in various malignancies, genuine extra-cranial RT are very rarely reported in adults [24–29], raising some doubts about diagnostic accuracy in such cases. In our hands, not all of the presumed adult RT are confirmed to be true “molecular” RT; such a distinction should have clinical implication, especially for the interpretation of adults phase I-II trials that aim to target RT vulnerability defined on pediatric tumors. However, we also describe adult RT that are not distinguishable from their pediatric counterparts. The clinical behavior of those tumors is also highly aggressive. Hence, one could suggest that adult patients with RT should be treated with pediatric protocols, but designs of pediatric trials for RT may not be adapted to older patients, who most often can't tolerate such high dose intensity. Nevertheless, all these tumors may respond similarly to biologically driven innovative therapy and, in that respect, common pediatric and adult RT early phase trials are justified. The underlying question remains the cells of origin of adult and children RT. We and others have proposed some early embryonic progenitors or stem cells as cells of origin [4, 5, 3]. The extreme rarity of true RT in adults may illustrate the highly restricted pool of originating cells after early childhood.

Altogether, our study helps defining RT among a wide series of SMARCB1-deficient cancers. Beyond these findings of potential interest for the understanding of RT biology, implementing this signature to technics available in daily routine on paraffin embedded tissue could now be set up. A gene-set based signature designed for quick and low cost profiling, similar to the one developed for medulloblastomas which has proven to meet clinical needs [32], could offer useful routine diagnostic tools and significantly help pathologists in their daily task.

## MATERIALS AND METHODS

### Inclusion criteria

All extra-cranial SMARCB1-deficient cancers referred to our laboratory for molecular analysis were included. SMARCB1-deficiency was defined by negative staining with BAF47 antibody. Altogether, 69 tumors could be included.

### Selection of tumors for the training set

In order to find a signature that could robustly discriminate RT from SD-NRT, we first intended to select tumors with ascertained diagnosis and compare them, within a “training set”.

At this aim, RT were restrictedly defined as tumors showing i) a proven *SMARCB1* biallelic inactivation, ii) with reviewed morphological features compatible with a diagnosis of RT, iii) without any other obvious diagnosis, iv) occurring before 5 years of age, v) in any location apart from the central nervous system and the clivus. All tumors included in the training set were reviewed by at least one of the expert pathologists in pediatric pathology (PF, DRV) and classified as RT. Sixteen tumors fulfilling these criteria could be identified and constitute the “RT” group for the training set (Supplementary Table 1).

For SD-NRT selected in the “training set”, diagnoses were the following: i) proximal type epithelioid sarcomas (ES, reviewed by JMC, DRV or PF): BAF47 negative tumors with epithelioid sarcoma phenotype, occurring after 10 years of age in the limbs, the thorax or the pelvis, with or without documented *SMARCB1* biallelic inactivation, ii) undifferentiated chordomas (reviewed by PV and DRV): BAF47 negative tumors occurring from the clivus and characterized by a high expression of “T” (Brachyury) by immunohistochemistry, with or without documented biallelic inactivation of *SMARCB1*, and iii) renal medullary carcinomas: defined as previously published [11], and showing a balanced translocation disrupting *SMARCB1*. Finally, 16 tumors were also identified for the training set (Supplementary Table 1). All other tumors (*n* = 37/69), without any clear diagnosis, constituted the “study cohort”.

### Assessment of *SMARCB1* status

Tumor DNA were extracted from frozen samples. All nine coding exons of *SMARCB1* were sequenced and large deletions were searched for by MPLA (Holland MRC kit), as previously described [30].

### Transcriptome analyses

Tumor RNAs were extracted using a miRNeasy mini kit (Qiagen ref: 217004). Labelled cRNA were hybridized on Affymetrix U133Plus2.0 arrays as described previously [31]. Gene expression data was normalized using gcRMA algorithm on custom Brainarray CDF. Differential subgroups were defined using two different methods: unsupervised hierarchical clustering (average linkage and Pearson correlation distance) and non-negative matrix factorization (NMF) (using NMF R package, with standard NMF algorithm method performed on 50 runs). The optimal number of subgroups have been defined on the lower proportion of ambiguous clustering value (PACk= CDFk(value index(max))-CDFk(value index(min))) and biological knowledge for consensus clustering method ; we also used the better cophenetic correlation score and biological knowledge for NMF method. All these methods are based on the expressed and variable genes obtained after elimination of background (background threshold: 3.5, fold change threshold: 1.2) and invariant genes using RIQR (threshold: 0.9; max(Q3-Q2, Q2-Q1)/Q2). Hierarchical clustering with silhouette was applied on gene sets which characterize differential subgroups by NMF. All clustering were performed using average linkage and Pearson correlation distance. Raw data are deposited with the following access number GSE94321.

### RT-PCR validation

We quantified the mRNA expression level of five different human genes (*DNMT3B*, *FGF9*, *FGFR2*, *HHIP*, and *HOTAIR*) in five RT and five SD-NRT to validate the expression data assessed by transcriptome micro-arrays. cDNA synthesis and subsequent qPCR reaction were performed on total RNA. We also quantified transcripts *TBP* as an endogenous RNA control gene. Real-time quantitative RT-PCR were performed using the ABI Prism 7900 Sequence Detection System (Perkin-Elmer Applied Biosystems) and the results were expressed as N-fold differences in target gene expression relative to the *TBP* control gene based on the 2^−ΔΔCT^ method. Primers for *TBP* and the target genes are available on demand.

### Exome sequencings

Whole Exome Sequencing (WES) was performed on 6 matched pairs of blood and tumors, and 6 tumors with no germline DNA, using Illumina Hi-Seq2500 leading to paired-ends 100×100bp with 100X expected coverage. Alignments were performed on human reference sequence (hg19) using Bowtie2-v2.1.0. Reads with mapping quality under 20 and reads that were marked as duplicates by Picard-v1.97 (http://broadinstitute.github.io/picard/) were excluded from further analysis. Variant calling was performed in parallel using 3 variant callers: Unified Genotyper and Haplotype Caller from GenomeAnalysisTK-v3.1.1 and Samtools-v0.1.18. Annovar-v2013-08-23 with cosmic-v65 and dbsnp-137 were used for the annotation and RefSeq for the structural annotation. Single nucleotide variants (SNVs) and InDels with a quality under 20, a depth of coverage under 10 or with less than 5 reads supporting the variant were filtered out. Then, coding variants reported in more than 1% of the population in the 1000 genomes (1000gAprl_2012), Exome Sequencing Project (ESP6500) or Exome Aggregation Consortium (ExAC version 0.3) have been discarded in order to filter polymorphisms. Finally, synonymous variants were filtered out. Whenever available, comparison of constitutional with somatic exome was performed. We conserved only variants that were heterozygous in somatic (0.1 ≤ x ≤ 0.8) and homozygous reference in all of our constitutional samples (x < 0.1 and depth ≤ 2) or homozygous alternative in somatic (x > 0.8) and heterozygous in constitutional (0.4 ≤ x ≤ 0.6); and which are not identified as benign, neutral, polymorphism or with low/no functional impact by the prediction tools (PP2 HID, PP2 HVAR, LRT, MutationTaster and MutationAssessor). For tumor samples without matched constitutional sample, we conserved i) variants which have COSMIC ID and ii) variants occurring in genes that we found mutated in tumor samples matched to their constitutional DNA. All variants have been confirmed using Integrative Genomics Viewer. We analyzed CNVs (copy number variants) using VarScan-v2.3.5 and DNA copy R package.

### Clinical data

Clinical data were retrospectively recorded from the local medical files. The collection of clinical data received the authorization from CCTIRS and CNIL (DR-2015-1994).

## SUPPLEMENTARY MATERIALS FIGURES AND TABLES








