# Supplementary material for: Embryonic signature distinguishes pediatric and adult rhabdoid tumors from other SMARCB1-deficient cancers
Source: Oncotarget. 2017 Mar 6;8(21):34245–57. doi: 10.18632/oncotarget.15939 (PMC5470964; doi:10.18632/oncotarget.15939)
Supplement: Supplementary file 1 [file oncotarget-08-34245-s001.pdf]

Embryonic signature distinguishes pediatric and adult rhabdoid tumors from other SMARCB1-deficient cancers

Supplementary Materials

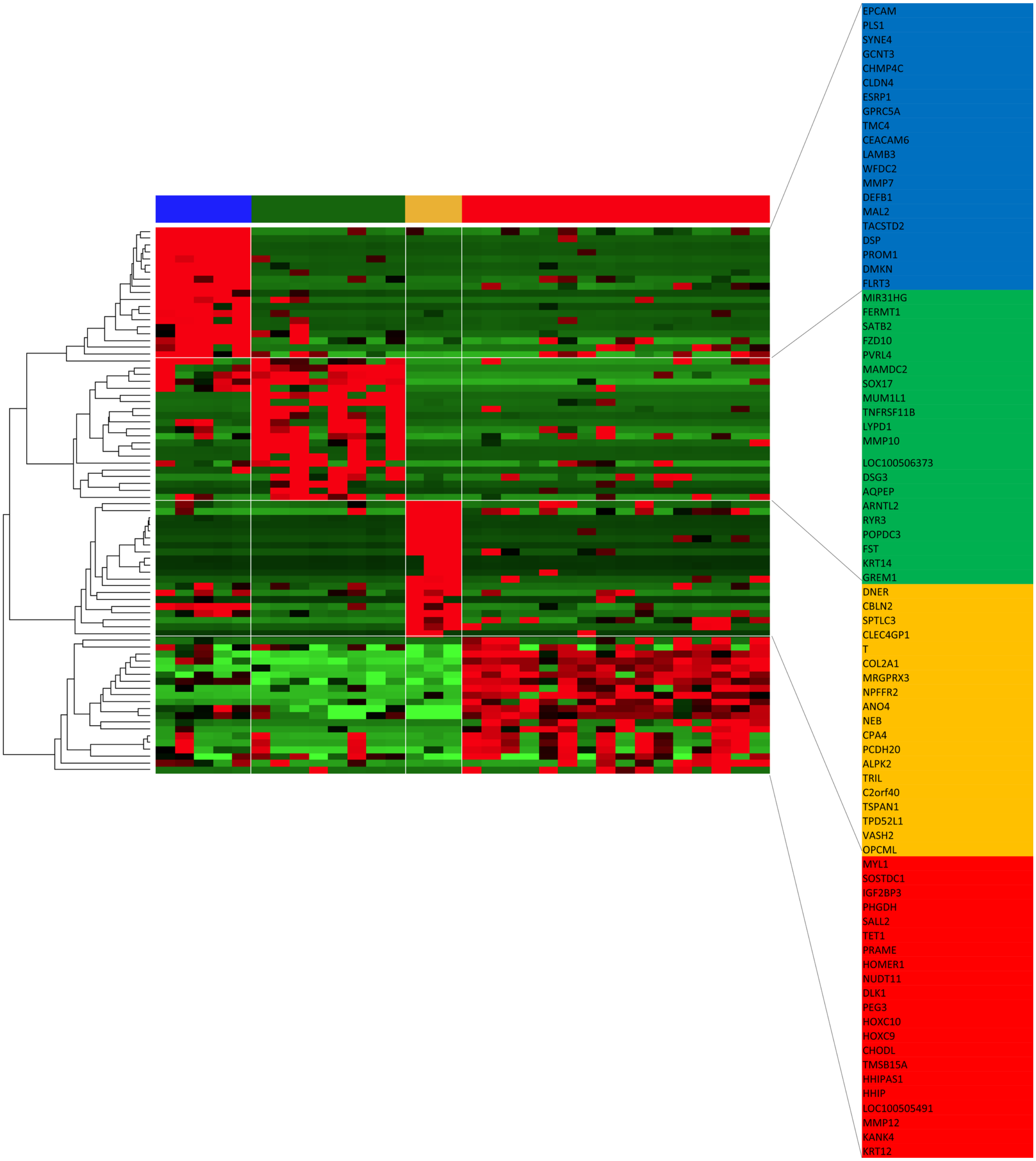

Supplementary Figure 1: Heat-map of training set tumors on a set of genes obtained by pair-wised analysis. RMC are indicated in blue, UC in yellow, ES in green and RT in red. The detail of genes used for these signatures is depicted on the right column.

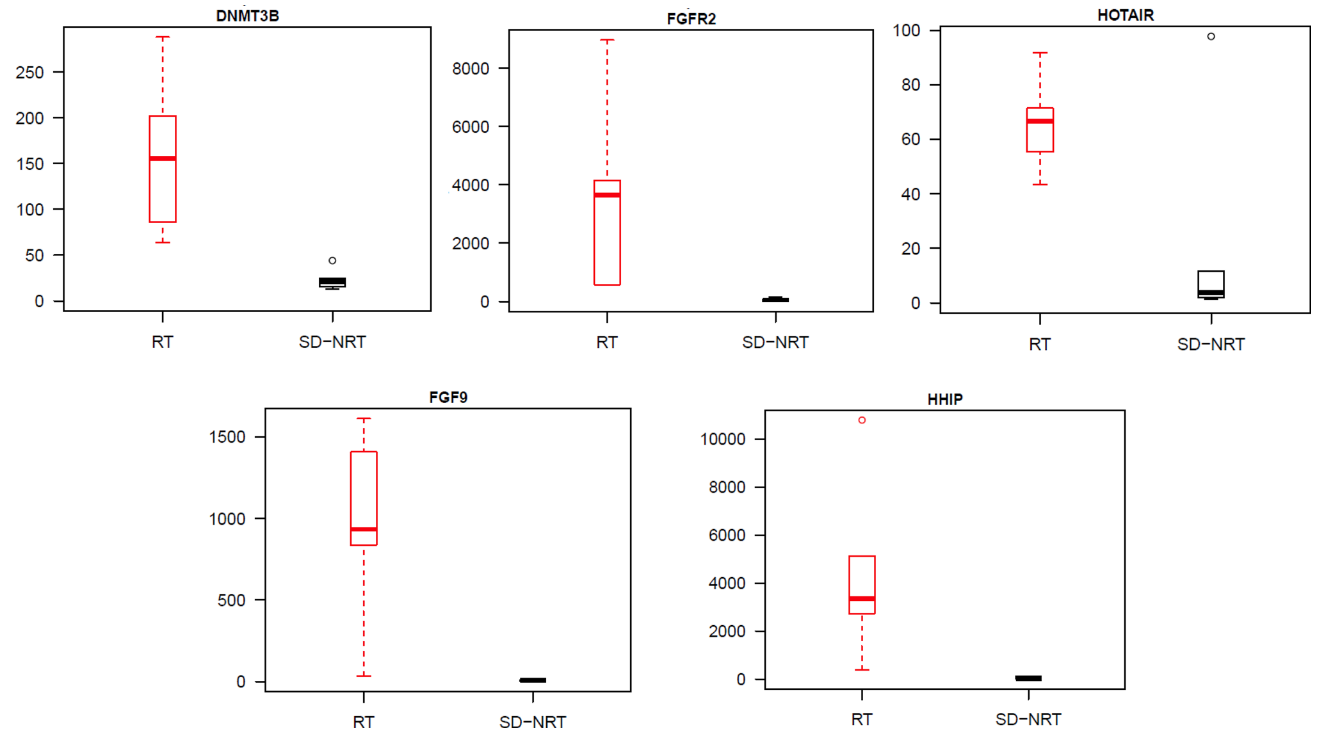

Supplementary Figure 2: RT-PCR confirmation on a short list of differentially expressed genes, in RT and SD-NRT. In y axis, expression level as N-fold differences in target gene expression relative to the *TBP* control gene based on the $2^{-\Delta\Delta CT}$ method.

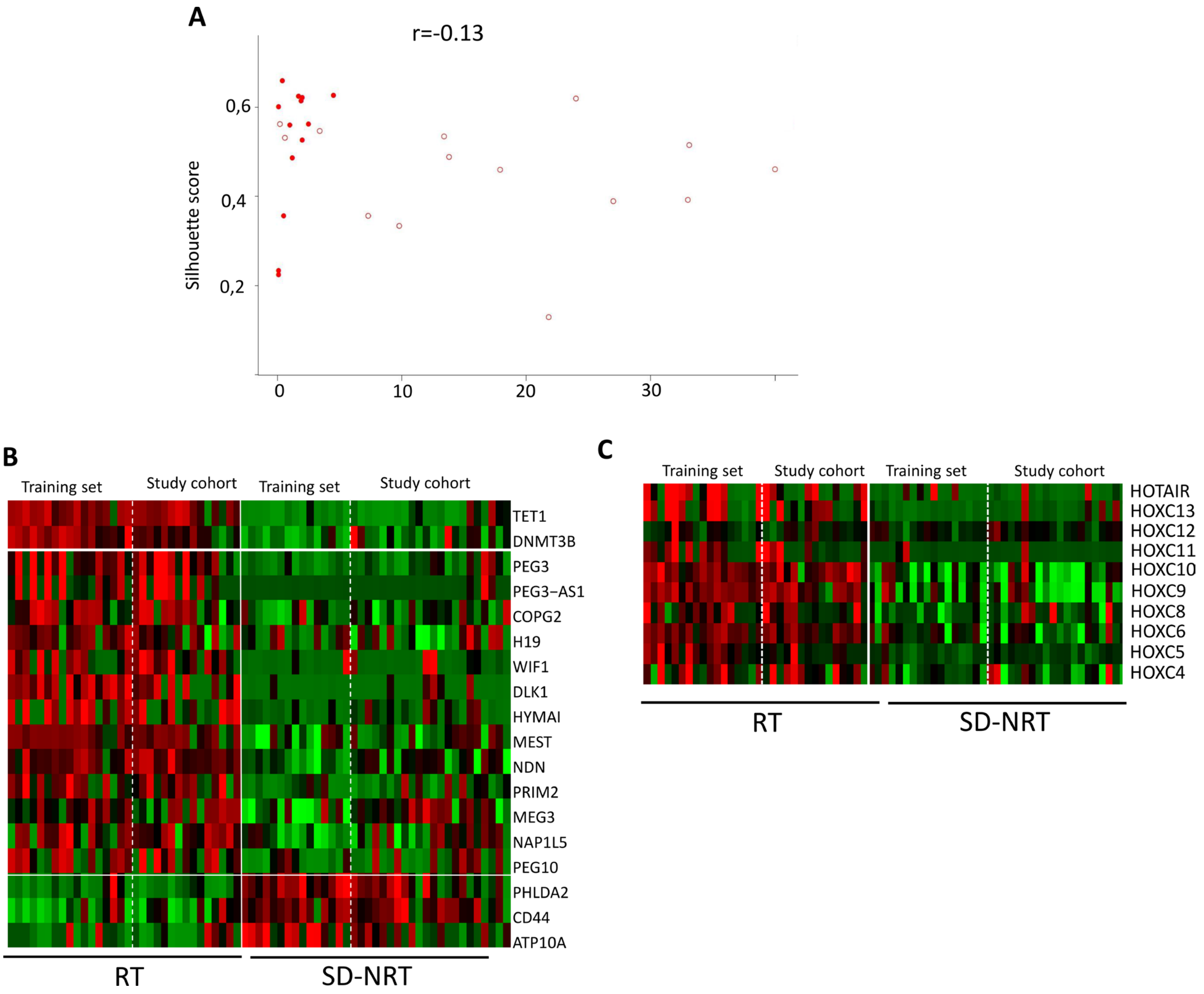

Supplementary Figure 3: Features of adult RT compared with pediatric RT. (**A**) Silhouette score is plotted according to age at diagnosis; full red circles and empty red circles represent RT from the training set, and the study cohort, respectively; no correlation nor anti-correlation (r = –0.13). (**B**) Heat-map on tumors from the training set and the study set showing imprinted genes; the list is defined by differential expression breween RT and SD-NRT in the training set; (**C**) Heat-map showing HOX clusters and HOTAIR HOX genes-regulator, in RT (i.e. RT from training set + study cohort tumors clustering with RT) and SD-NRT (i.e. SD-NRT from training set + study cohort tumors clustering with SD-NRT)

Supplementary Table 1: Clinical outcome of late-onset RT

	Meta.	Size	Delay from first symptoms	Chemotherapy regimen	Surgery	Radiotherapy In Grays	Best response
**RT**							
INI44	NA	NA	NA	NA	NA	NA	NA
INI61	M0	> 5 cm2	NA	CyCE<br>VDCy	R1	54Gy	CR
INI64	M+<br>Soft tissue, bones	NA	1 year	CAdO/VP-CBP<br>Nvb-CPM	None	20Gy	PD
INI105	M0	> 5 cm2	NA	None	R1	None	PR
INI120	M0	> 5 cm2	3weeks	CyCE<br>VAC	R0	45Gy	CR
INI127	N+	< 5 cm2	6 months	AI x6	None	44Gy	CR
INI135	M0	> 5 cm2	NA	NA	NA	NA	NA
INI136	M0	> 5 cm2	8 months	CyCE<br>VDCy	None	54Gy	PR
INI143	M0	> 5 cm2	2 weeks	CyCE<br>VDCy	R0	45Gy	CR
INI174	M+<br>Bone	> 5 cm2	2 months	VP16-Ifo<br>3x Dox<br>VP-CPM	0	0	PR
INI176	M+<br>Soft tissue	> 5 cm2	NA	NA	NA	NA	NA
INI185	NA	NA	NA	NA	NA	NA	NA
**SD-NRT**							
INI20	M+<br>Bone	> 5 cm2	2 months	VP16-CBP<br>ICE, CAdO	None	None	PD
INI37	N+	> 5 cm2	6 weeks	AE<br>ICE	None	30Gy	PD
INI65	M+<br>Lung	> 5 cm2	>12 months	Unknown	R2	None	PD
INI86	NA	NA	NA	NA	NA	NA	NA
INI114		> 5 cm2	11 months	NA	NA	NA	NA
INI115	M0	< 5 cm2	NA	None	R0	60Gy	CR
INI117	M0	> 5 cm2	1 month	VP16	R2	None	PD
INI123	M+<br>Liver	> 5 cm2	NA	DVCP<br>MAID	None	None	SD
INI128	M0	NA	NA	NA	NA	NA	NA
INI129	NA	NA	NA	NA	NA	NA	NA
INI130	M0	> 5 cm2	3 months	Ifo-Doxo	R0	41Gy	CR
INI131	M0	> 5 cm2	NA	CDDP-PMT<br>VCR-Doxo	None	None	PD
INI132	M0	> 5 cm2	NA	HD MTX<br>CHOP	R0	60Gy	CR
INI133	M0	> 5 cm2	3 months	Ifo-Doxo	R0	50.4Gy	CR
INI134	N+	> 5 cm2	NA	None	None	None	PD
INI152	Bone,<br>Lung	> 5 cm2	1 month	Doxo	None	None	PD
INI175	M0	> 5 cm2	3 months	VDCy<br>CyCE	R1	55Gy	PR
INI182	M+<br>lung	> 5 cm2	2 months	NA	R0	None	SD

Meta.: metastatic status NA: data not available. M0: no metastasis. N+: lymph node involvement. M+: other metastasis. VIDE: Vincristine, Ifosfamide, Doxorubicine, Etoposide. CyCE: Cyclophosphamide, Carboplatin, Etoposide. VDCy : Vincristin, Doxorubicin, Cyclophosphamide. Doxo : doxorubicin. Ifo : Ifosfamide. VP16-CBP : Etoposide, Carboplatin. CAdO : Cyclophosphamide, Doxorubicin, Vincristin. VAC : Vincristin, Actinomycin, Cyclophosphamide. CDDP-PMT : Cisplatin, Pemetrexed. HD MTX: Methotrexate, High doses. CHOP: Cyclophosphamide, Doxorubicin, Vincristin, Prednisone. R2: macroscopic residue. R1: microscopic residue. R0: no residue. PD: progressive disease. SD: stable disease. PR: partial response. CR: complete response.

Supplementary Table 2: Differentially expressed genes and pathways, SDNRT vs RT. See Supplementary_Table_2

Supplementary Table 3: NMF signature for all SMARCB1-deficient tumor types. See Supplementary_Table_3

Supplementary Table 4: List of significant Single Nucleotide Variants and InDels. See Supplementary_Table_4
